# A Systematic Review of Mental Health Professionals, Patients, and Carers’ Perceived Barriers and Enablers to Supporting Smoking Cessation in Mental Health Settings

**DOI:** 10.1093/ntr/ntac004

**Published:** 2022-01-08

**Authors:** Lisa Huddlestone, Emily Shoesmith, Jodi Pervin, Fabiana Lorencatto, Jude Watson, Elena Ratschen

**Affiliations:** Department of Health Sciences, University of York, York, UK; Department of Health Sciences, University of York, York, UK; Department of Health Sciences, University of York, York, UK; Centre for Behaviour Change, University College London, London, UK; Department of Health Sciences, University of York, York, UK; Department of Health Sciences, University of York, York, UK

## Abstract

**Introduction:**

Evidence-based smoking cessation and temporary abstinence interventions to address smoking in mental health settings are available, but the impact of these interventions is limited.

**Aims and Methods:**

We aimed to identify and synthesize the perceived barriers and enablers to supporting smoking cessation in mental health settings. Six databases were searched for articles reporting the investigation of perceived barriers and enablers to supporting smoking cessation in mental health settings. Data were extracted and coded using a mixed inductive/deductive method to the theoretical domains framework, key barriers and enablers were identified through the combining of coding frequency, elaboration, and expressed importance.

**Results:**

Of 31 included articles, 56 barriers/enablers were reported from the perspectives of mental healthcare professionals (MHPs), 48 from patient perspectives, 21 from mixed perspectives, and 0 from relatives/carers. Barriers to supporting smoking cessation or temporary abstinence in mental health settings mainly fell within the domains: environmental context and resources (eg, MHPs lack of time); knowledge (eg, interactions around smoking that did occur were ill informed); social influences (eg, smoking norms within social network); and intentions (eg, MHPs lack positive intentions to deliver support). Enablers mainly fell within the domains: environmental context and resources (eg, use of appropriate support materials) and social influences (eg, pro-quitting social norms).

**Conclusions:**

The importance of overcoming competing demands on staff time and resources, the inclusion of tailored, personalized support, the exploitation of patients wider social support networks, and enhancing knowledge and awareness around the benefits smoking cessation is highlighted.

**Implications:**

Identified barriers and enablers represent targets for future interventions to improve the support of smoking cessation in mental health settings. Future research needs to examine the perceptions of the carers and family/friends of patients in relation to the smoking behavior change support delivered to patients.

## Introduction

There are substantial inequalities in morbidity and premature mortality between individuals with mental health problems and those without.^1^ One of the largest contributory factors to early mortality in this population is smoking.^2^ Among individuals with a common mental health condition in England, smoking prevalence is over 50%,^3^ compared with 14% in the general population,^4^ and this difference increases further for more severe mental health conditions.^5^

While the number of smokers in the general population has been steadily declining over recent decades,^6^ the number of people with mental health conditions who smoke have not been declining at the same rate.^7^ Those with mental health conditions are more likely to display patterns of heavy smoking,^3,5,8^ greater dependence on nicotine, and more severe withdrawal symptoms when quitting, and lower quite rates.^9–12^ Previous research has estimated the percentage of smokers with mental health conditions vary dependent on setting, but can reach up to 70% of inpatients smoking.^13,14^ Yet, smokers with mental health conditions are equally, or more motivated to quit smoking than those without mental health conditions.^15^ However, they are less likely to receive support compared with the general population.^16^ There are many reasons for this, including the smoking culture within mental health services,^16,17^ often driven by misconceptions, for example, relating to the “therapeutic” function of smoking in this population.^18^

Despite this, the World Health Organisation recommends that all healthcare facilities are smokefree, a policy that is increasingly being adopted internationally.^19^ However, the healthcare system and the respective development and implementation of tobacco control policies can vary substantially across countries.^20^ These differences may present various contextual, political, and economic barriers that may impact on the success of quitting behaviors that require separate consideration. For example, economic barriers may limit the potential to implement evidence-based smoking cessation interventions in healthcare settings,^21^ and there is a wide variation in the provision of smoking cessation advice offered by healthcare professionals dependent on setting.^22^ Help-seeking behavior may also differ by setting and influence smoking-related outcomes. Previous literature has reported individuals living in higher-income countries were more likely to seek advice from a healthcare professional to quit, and have higher use of quit smoking medications, compared with those in low- and middle-income countries.^22^ As such, there are considerable differences between settings in regard to quitting behaviors and type of support used. Such variation reflects the differences in tobacco control implementation, the capacity of the country and the priority given to specific policies (eg, regulatory measures and the provision of cessation support).^22^

Regardless of setting, the evidence base reports that there are factors that can influence the success of quitting behaviors among vulnerable groups.^23,24^ Within health behavior literature, factors that hinder an individual from making a health behavior change have been referred to as barriers, and factors that facilitate an individual to make a change are referred to as enablers. Barriers and enablers can be conceptualized as either individual or structural psychosocial factors.^25^ Individual factors refer to subjective experience, and can be nonmodifiable (eg, age, ethnicity, nicotine dependence), whilst others are modifiable and thus, potentially amenable to intervention (eg, plans to not smoke or a desire to quit).^26^ Structural factors include organizations and the relationship between these organizations and individuals. Likewise, some are nonmodifiable (eg, pharmacist’s behavioral control of reconciling medications),^27^ whilst others are modifiable (eg, accessibility to smoking cessation interventions).^28^

Despite a growing evidence base in relation to barriers and enablers to the implementation of behavior change interventions by healthcare professionals,^29^ there remains a lack of focus on those factors that are shared across professional groups.^30^ Given this limitation, it is important to differentiate between the roles that individuals involved in the delivering and receiving of smoking cessation interventions may play. For example, clinical staff are likely to be involved in the implementation and delivery of the intervention, and thus, the perceived lack of time is a frequently reported barrier,^31,32^ whereas nonclinical staff may report barriers at the commissioning and policy level (eg, lack of adequate information on the cost, volume, and quality of healthcare services).^33^

In addition to individual and organizational factors, barriers and enablers may also be conceptualized as socially influenced. For example, the family is an influential context in which smoking behavior occurs.^34^ Such social networks may play an important role in the individual’s quit attempt, since cohabitants smoking status is a known major determinant for adult smoking behavior change.^35,36^ Indeed, previous research reports cases of family members actively discouraging quit attempts by people with mental illness, as well as encouraging the maintenance of smoking due to concerns about cessation adversely impacting the individual’s mental health^37^ or because smoking was perceived to be the individual’s only source of enjoyment.^38^ However, and somewhat paradoxically, research also reports that family relationships are a prime motivator to quit,^38^ indicating that family may also be a crucial enabler for smoking cessation.

Understanding these perceived barriers and enablers to quitting is important in order to facilitate our understanding of smoking, relapse and quitting-related behaviors, to inform appropriate policy, and to facilitate the development of more effective tailored smoking cessation interventions. Furthermore, due to the increased prevalence and overall reduced rates of successful cessation success among those with mental health conditions,^9–12^ a need identify the barriers and enablers to quitting smoking in mental health settings from the perspective of people with mental illness and mental health professionals and those providing mental health services is required.

Effective behavior change interventions require an understanding of the broader context of the problem (eg, the social and environmental context, and noncontextual influences on behavior such as knowledge consequences and motivation).^39^ The theoretical domains framework (TDF) is an integrative theoretical model that synthesizes main behavior change constructs across key theories into 14 domains, such as knowledge or goals.^40^ The TDF is helpful for investigating implementation barriers and enablers, and provides a useful conceptual basis for assessing implementation problems, designing interventions to enhance healthcare practice, and understanding behavior change processes.^29^

Therefore, the aim of this systematic review was to identify and synthesize the evidence relating to the barriers and enablers that influence smoking abstinence, and the delivery of smoking cessation or temporary abstinence interventions in mental health settings from the perspective of those delivering and receiving such interventions. Specifically, the research questions are:

1. What are the modifiable barriers and enablers that influence smoking cessation or temporary abstinence for patients in mental health settings?
2. What are the modifiable barriers and enablers that influence the delivery of smoking cessation or temporary abstinence interventions for mental health professionals (MHPs) in mental health settings?
3. What are the modifiable barriers and enablers that influence the support of smoking cessation for relatives/carers in mental health settings?

## Methods

### Search Strategy

The systematic review was conducted in accordance with PRISMA guidelines and registered on PROSPERO (CRD42020193125).

Searches were conducted in four bibliographic databases (MEDLINE, EMBASE, PsycInfo, and CINAHL), as well as the Cochrane Central Register of Clinical Trials, and the UK Clinical Research Network Portfolio database. The search strategy included search terms relating to the population (eg, inpatients, mental health nurses, relatives/carers), intervention (smoking cessation or temporary abstinence), outcome (eg, barriers, enablers), and relevant settings (eg, mental health services). Supplementary Table 1 provides details of the search terms. Searches were limited to papers published in English, and from 1990 onwards due to pharmacological, behavioral, and other counseling approaches not being widely available prior to 1990.^41^

### Inclusion and Exclusion Criteria

Article inclusion were based on the population, intervention, comparator, outcome (PICO) method for eligibility, shown in Table 1. Articles utilizing quantitative experimental (including randomized control trials) or observational methods, qualitative methods, or mixed-methods were eligible for inclusion. Systematic reviews, conference papers, or those articles that were not original research were excluded.

**Table 1.** Criteria for Article Inclusion Based on the PICO Method for Eligibility

Population	• Adult smokers using community, outpatient, and acute inpatient mental health services and their family, friends, carers, and visitors
	• Members of staff working inpatient and outpatient, or community mental health settings
Intervention	• Smoking cessation (including cutting down to quit)
	• Temporary abstinence (in the context of an inpatient admission)
	• Interventions aimed at promoting cessation or preventing relapse after temporary abstinence/quitting (eg, in the context of discharge from an inpatient admission)
Comparator	Not applicable
Outcome	• Reported barriers to and enablers of the use, implementation, and delivery of evidence-based smoking cessation interventions
	• Other influences on the use and uptake of interventions may include type of provider, specification of pathways, type of intervention (eg, frequency, duration), and intended and unintended consequences of interventions

### Data Screening

Endnote X9 was used to record publications at all stages of the selection process. After removal of duplicates, two members of the research team (LH and ES) independently screened all identified titles and abstracts against the inclusion and exclusion criteria to ensure consensus. A third author (JP) rescreened 100 titles and abstracts to ensure reliability. Where disagreements arose, these were settled by discussion. Where exclusion could not be determined from the abstract, articles were included for full-text review. Full-text copies of potentially eligible studies were obtained and a final decision was made on inclusion by consensus among the review team.

### Data Extraction

Data were extracted using a customized spreadsheet by three authors (LH, ES, and JP). The extracted study characteristics were country, research design, methods, setting (inpatient, outpatient, community), participants, and target behavior (smoking cessation or temporary abstinence for patients; delivering smoking cessation support for MHPs). Authors identified and extracted quantitative and qualitative data reporting perceived barriers and enablers associated with target behaviors.

### Quality Assessment

Article quality was assessed using the Mixed Methods Appraisal Tool (MMAT).^42^ Two authors (ES and JP) rated independently rated included studies, and a third author (LH) independently assessed a random sample of 11 (35%) studies. Minor differences in opinion relating to the quality of studies were resolved through discussion.

### Data Analysis

In order to identify and understand the context of barriers and enablers to smoking behavior change, the TDF was utilized. The approach to data analysis followed the combined three-step method reported by Graham-Rowe et al.,^43^ in which content and framework analysis approaches are combined:

1. Deductive content analysis was conducted by coding the extracted data to the TDF. Two authors (LH and ES) coded the extracted data from all studies according to which domain they were judged to best represent. For example, the extracted data point barriers that were notably endorsed by psychiatrists were “lack of time (49%)” ^44^ would be coded to the domain “environmental context and resources,” and “social norms, attitudes and behaviors toward smoking as an undesirable social behavior helped some participants in the quitting process” ^45^ would be coded to the domain “social influences.” Coding was guided by the definitions of the TDF domains outlined by Cane et al.^40^ Three authors (LH, ES, and JP) reviewed and verified each coded item.
2. Inductive thematic synthesis was conducted to combine similar data points coded to the same domain, and inductively generating a summary theme label and corresponding subthemes. Coding was conducted independently by two authors (LH and ES), with discussion to identify consistency in the development of themes and subthemes. Discrepancies between coders were resolved through discussion with a third author (JP). Themes were then categorized as either a barrier, enabler, or mixed influence, and as relating to the perception of patient, carer, family member, friend, MHP, or organization.
3. Key barriers and enablers were then identified by ranking TDF domains in terms of importance using established criteria^46^: (1) frequency (number of studies that identified each domain); (2) elaboration (number of thematic subthemes and themes) within each domain; and (3) expressed importance (a statement from the authors’ discussion or direct quotations from the study participants expressing importance). The frequencies from each of the three categories were combined and a median frequency with standard deviation was calculated. TDF domains exceeding this calculated mean frequency were considered as being of importance.

## Results

### Description of Studies

**Figure 1.**
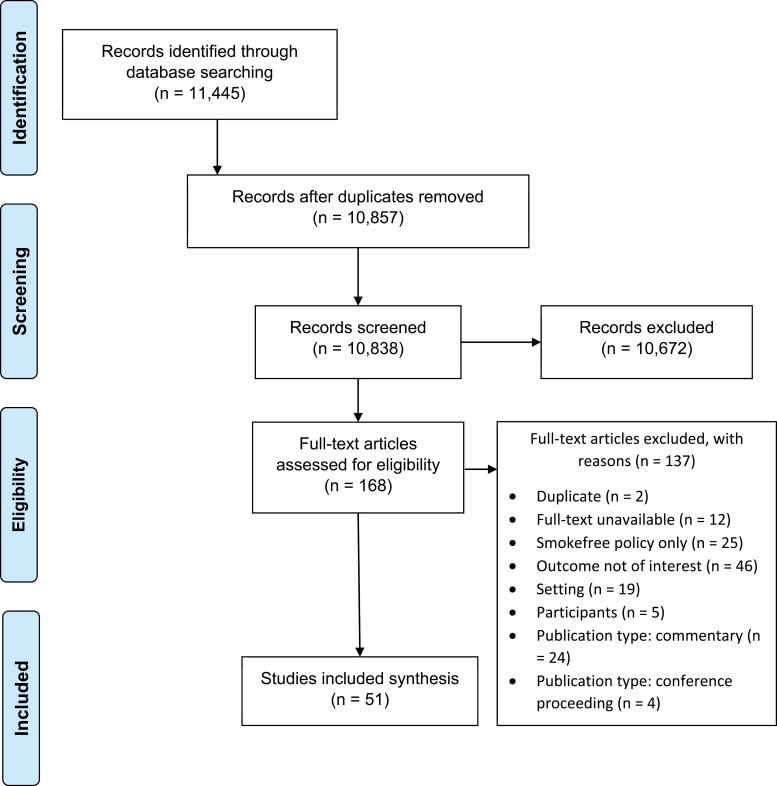
Preferred Reporting Items for Systematic Reviews and Meta-Analyses (PRISMA) flow diagram.

Database searches yielded a total of 11 445 articles. After the removal of duplicates and screening of titles, abstracts, and full-text articles, 31 papers were included in the review^31,44,45,47–74^ (Figure 1).

Fourteen studies were observational quantitative studies, eight utilized a qualitative methodology, six were randomized control trials, and three adopted a mixed-methods design. A total of 8626 participants were recruited across 30 of the included studies, with one quantitative study not reporting a sample size.^69^ Most studies were conducted in community mental health settings (*n* = 12), followed by inpatient settings (*n* = 9), and outpatient clinics (*n* = 5). A number of studies gathered data in mixed mental health settings (*n* = 5). Seventeen studies recruited only patients, seven studies recruited a range of clinical and nonclinical MHPs, five included both patients and MHPs, and two obtained the perceptions of mental health service managers and directors). Studies recruiting carers, family members, or friends of individuals with mental health problems could not be identified. Full study characteristics are presented in Supplementary Table 2.

### Quality Assessment

All studies clearly stated their research questions or research objectives. The majority provided the requisite information required by the MMAT. Those which lacked the required information included four randomized control trials, where it was not possible to ascertain the appropriateness of randomization^73^ and blinding procedures,^48,51,59,73^ and three quantitative descriptive studies lacked sufficient information to assess the risk of nonresponse bias.^61,62,69^ All studies used established methods that were appropriate to answer the research questions. Full detail of the included studies is provided in Supplementary Table 3.

### Frequency of Identified Barriers and Enablers to the Delivery and Receipt of Smoking Behavior Change Interventions

A total of 75 barriers and 50 enablers were identified across the included articles. Fifty-six barriers and enablers were elucidated from the perspectives of MHPs or organizations (44 barriers; 12 enablers), and 48 from the perspective of patients (17 barriers; 31 enablers). Twenty-one were from a mixed (patient/MHP/organizational) perspectives (14 barriers; 7 enablers).

Barriers and enablers were identified across 13 of the 14 TDF domains. The majority of these fell within the domains environmental context and resources (*n* = 20 barriers; 9 enablers); knowledge (*n* = 15 barriers; 5 enablers); intentions (*n* = 10 barriers; 5 enablers); and social influences (*n* = 7 barriers; 7 enablers).

Supplementary Table 4 presents the themes inductively generated for each TDF domain, organized by perspective (patient, MHP, organization, mixed), and influence (barrier, enabler, mixed). Supplementary Table 5 also presents all the themes and subthemes generated in the 13 identified domains of the TDF, organized by perspective (patient, MHP, organization, mixed), and influence (barrier, enabler, mixed).

### Identification of Important TDF Domains

#### Frequency of Coding to Domains

Data were coded most frequently to the domains of: environmental context and resources (*n* = 16 articles); knowledge (*n* = 12 articles); social influences (*n* = 10 articles); intentions (*n* = 9 articles); beliefs about capabilities (*n* = 8 articles); and emotion (*n* = 7 articles).

#### Level of Elaboration

The level of elaboration was calculated from the number of themes and subthemes generated within each domain identified in the inductive analysis. Environmental context and resources had the highest number of themes (*n* = 3) and subthemes (*n* = 23), followed by intentions and emotion (*n* = 2 themes; 9 subthemes in each domain), and social influences and knowledge (*n* = 2 themes; 8 subthemes in each domain).

#### Importance Expressed by Study Authors

Fifteen authors interpreted study findings as identifying important influences. Important domains were: environmental context and resources (15 items in 16 studies); knowledge (9 items in 12 studies); social influences (10 items in 10 studies); intentions (8 items in 9 studies); and emotion (7 items in 7 studies).

#### Ranking Criteria Convergence

**Figure 2.**
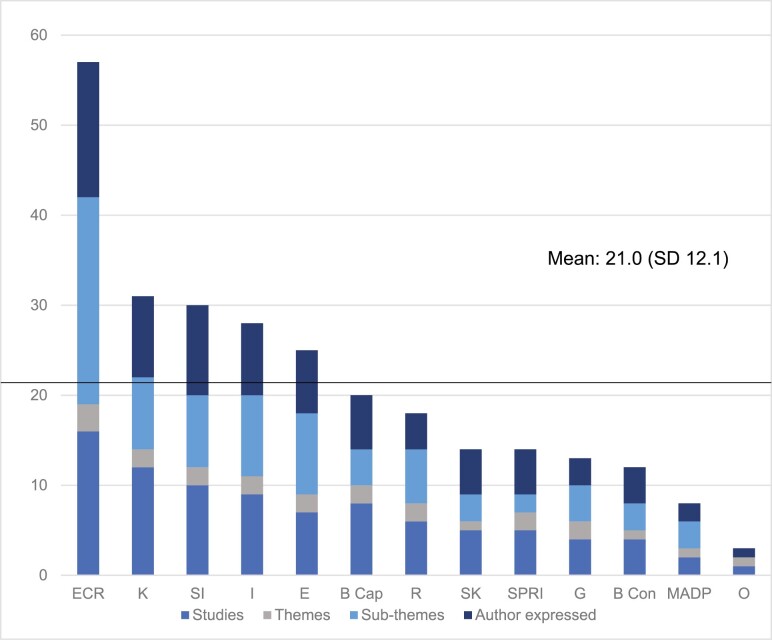
Domains ranked according to the importance criteria of frequency, elaboration, and expressed importance. ECR = environmental context and resources; K = knowledge; SI = social influence; I = intentions; E = emotions; BCap = beliefs about capabilities; R = reinforcement; SK = skills; SPRI = social/professional role identity; G = goals; BCon = beliefs about consequences; MADP = memory, attention, and decision processes; O = optimism.

Domains ranked according to the importance criteria of frequency, elaboration, and expressed importance are presented in Figure 2. Accordingly, the most important domains were: environmental context and resources, knowledge, social influences, intentions, and emotion. These are summarized narratively below.

#### Environmental Context and Resources

Overall, environmental context and resources appeared to have a mixed influence on the delivery and receipt of interventions to support smoking cessation or temporary abstinence following discharge from a mental health setting. The theme “integration of services” related to the organization and cooperation within and between mental health and other health services, and was reported by both patients, MHPs, and organizations. For many of these participants, “integration of services” identified barriers concerning the absence or cohesion of referral and smoking cessation support pathways, and the availability of resources.^31,44,47,63^

The theme of “presence or absence of available support” related to the availability and accessibility of a range of preference-based support, and the materials and format of the support. For example, these barriers included access to nicotine replacement therapy while admitted to a smokefree mental health setting, as well as the inaccessibility of nicotine replacement therapy due to financial costs following discharge.^55,61,62^ Moreover, MHPs reported that resources were not adequate (eg, lack of referral and/or clinical resources), and this negatively impacted the implementation of either the smokefree policy or the smoking cessation support available.^44,56,61,70^ In terms of the support materials, MHPs and patients perceived the format of the support materials as an enabler, if they were easy to use, colorful, and incorporated useful information.^52^ However, a barrier would include potential literacy issues for some patients, but this could be overcome with the additional use of technology, if resources were available.^52^

Finally, the theme of “task rich and time poor” exemplified the perceived competing demands on MHP’s time and resources as a barrier to the delivery of smoking cessation interventions. Competing demands included: limited clinical time to address mental health needs and tobacco use, the need to prioritize the support offered individually to patients, and immovable organizational and service-level responsibilities.^44,50,60,61,63^

#### Knowledge

Several studies reported a lack of awareness about tobacco use, its links to mental illness, and treatment both during admission and within the community as barriers to both the delivery and receipt of interventions.^31,47,52,55,56,60,62,63,70^ Lack of knowledge and misinformation was widespread across both groups. For example, one study identified that interactions around smoking that did occur were ill informed in relation to inaccurate advice.^52^ Another study identified a lack of knowledge and information with regard to strategies to support stopping smoking, especially the use of nicotine replacement therapy products.^55^ Lastly, MHPs were found to actively discourage smoking cessation attempts due to concerns about the impact on patients’ mental health or due to a perception that stop smoking medications are unsuitable for people with a mental health condition.^63^

Conversely, when MHPs were perceived to have a greater awareness and knowledge regarding tobacco use and its links to mental illness, this was perceived as an enabler to patient engagement.^63^ Additionally, patients identified access to a wide range of information as an enabler (eg, more detailed information about the health consequences, social impact of smoking, and pharmacological support).^49,51,52^ Thus, providing training and education was identified as a crucial component by both MHPs and patients, including evidence-based pharmacotherapies and behavioral interventions.^56^ One study reported that both patients and MHPs acknowledged the importance of education about the harmful effects of tobacco use versus the potential benefit of symptom control.^56^

#### Social Influences

A mixture of barriers and enablers were identified within the domain of social influence, all from the perspective of the patients. The theme “influence of social network members” captures the smoking norms, attitudes and behaviors of social network members, and how these impacts on the individual. A number of patients reported that smoking was normative in many social contexts, and as a result, quit attempts were challenging due to their peers and family smoking around them.^45,55,58^ Conversely, when social network members considered smoking an undesirable behavior, this helped some patients in their quit attempt.^45,55,57^ One study identified that almost all patients (92%) could identify a key support person in their life on whom they could rely on to provide assistance and general support, and 70% of participants with a partner believed the partner would be supportive of them making a quit attempt.^66^

The theme “smoking culture within a mental health context” highlights that many patients identified the smoking culture as a barrier. For example, frequently observing tobacco use among MHPs and other patients challenged one’s own quit attempt.^52,56^ One study reported that when social activities were available, these reinforced smoking behaviors. Indeed, patients frequently commented on how difficult it was to consider quitting when those around them smoked. Both MHPs and patients viewed smoking as a social event, and a way to connect with family, peers, and staff.^56^ However, another study reported that some MHPs acknowledged how smoking was once an activity shared between staff and patients, but the Trust had progressed in denormalizing the social culture that was once ingrained into the mental health context.^31^

#### Intentions

The theme “stability of intentions and stages of change” relates to the patient’s intentions and their readiness to quit. Many patients were determined and motivated to quit, and had intentions to do so, despite a potential lack of self-belief in their ability.^57^ One study did report that measures of motivation (stages of change, thoughts about abstinence scales) predicted abstinence status significantly,^59^ indicating that positive intentions are an enabler for smoking cessation. A number of studies identified that MHPs perceived patient’s lack of intention or interest to quit as a barrier for the individual to engage with smoking cessation support.^44,50,60,61^

Lastly, one study reported on the lack of intention of MHPs to deliver smoking cessation interventions. For example, nursing staff had lower scores than medical staff with regard to the intention to provide tobacco treatment.^67^ The findings reported that staff attitudes were independently associated with intentions to provide tobacco treatment.^67^

#### Emotion

All of the data coded to the domain of emotion was identified as barriers to the delivery and receipt of interventions to support smoking cessation or temporary abstinence. The theme “coping mechanisms for stress” highlighted that smoking was often used to cope with acute stressors (eg, health scares, bereavements), everyday stresses of life, and also as a coping mechanism specifically in relation to one’s mental health diagnosis.^52,56–58,62^ One study reported that the majority of MHPs agreed at least in part with the statement that “smoking relieves efficiently from daily tensions or stress.” ^62^

In addition, the theme “lack of meaningful activities” was predominantly identified as a barrier by patients, and frequently referred to boredom, inactivity and time alone that would subsequently lead to smoking behavior as an activity to fill time or manage cravings.^52,55–57^ Furthermore, one study reported that boredom was common among patients in both hospital and community settings, and individuals maintained they smoked in the absence of other meaningful daily activities.^56^

## Discussion

This paper presents a systematic, theoretically informed approach to the identification of perceived barriers and enablers to supporting smoking cessation in mental health settings. Our findings identify five TDF domains as being important influences on delivery or receipt of smoking cessation or temporary abstinence support: (1) environmental context and resources, (2) knowledge, (3) social influences, (4) intentions, and (5) emotion.

This systematic review emphasizes the need for smoking cessation support for people with mental health conditions to be integrated within and between mental health and other health services. Many of the factors identified by MHPs as barriers to addressing smoking in mental health settings link directly to the environmental context and resources. For example, the importance of integration of services, and overcoming competing demands on staff time and resources.^44,50,60,61,63^ These findings emphasize the importance of a protected space with allocated time to focus on smoking cessation support outside of routine work in mental health settings.^63^ Furthermore, such findings are also acknowledged by other researchers who highlight that people with mental health conditions can be disadvantaged by fragmented care.^75^ Likewise, authors have also demonstrated that if MHPs were provided with protected space and time to focus on smoking cessation, they were able to effectively liaise between primary and secondary services.^63^

Similarly, through the identification of the barriers concerning the absence or cohesion of referral and smoking cessation support pathways, the need to consider the additional challenges that people with mental health conditions encounter when undertaking cessation attempts is indicated. Indeed, authors note that the variability and complexity of mental health service provision may result in confusion for patients, particularly when they are required to self-refer to cessation services following discharge from a mental health inpatient setting.^76^ Lastly, attention is drawn to the importance of available and accessible preference-based support. Moreover, in alignment with previous research, the personalization of support within a mental health context has been shown to enable changes in smoking behavior.^73^ Indeed, results from the SCIMITAR+ trial confirm the positive influence of bespoke smoking cessation interventions in this population, finding a doubling of the likelihood of quitting at 6 months in comparison to the control group.^77^

Within the TDF domain of “knowledge,” a lack of awareness and comprehension was frequently reported in relation to tobacco use, its links to mental illness, and the support available.^31,47,52,55,56,60,62,63,70^ What is more, MHPs who receive specialist training to offer services designed to improve an individual’s mental health have a crucial role in reducing tobacco smoking among people with mental health conditions, as they are best placed to encourage and support smokers to quit.^78^ However, the findings from this review highlight a need for increased specialist training in smoking cessation interventions, as well as broader education to challenge misconceptions about smoking cessation in the context of mental illness and mental health services. Additionally, improved access to flexibly delivered mandatory training (with periodic refreshers) for MHPs should improve the consistency of smoking-related health messages delivered to patients. Correspondingly, previous research advocates for additional training of smoking cessation advisors in the United Kingdom working with people with mental health conditions.^79^

The TDF domain of social influence appeared to have mixed consequences on smoking cessation in mental health settings, and patients frequently identified support networks as either a barrier or an enabler. These findings are consistent with a social norms perspective on health behavior change, whereby individual choices are significantly influenced by the behaviors and opinions of important others.^80^ Awareness of ex-smokers and those within a patient’s social network who are also undertaking quit attempts may be particularly important for populations that experience a high prevalence of smoking.^28,81^ Thus, the exploitation of patients wider social support networks may be an effective strategy for supporting smoking cessation among individuals with mental health conditions.

Despite intentions of the authors to understand the barriers and enablers to addressing smoking in people with mental health conditions from the perspective of their carers, family members, or friends, the included studies did not yield evidence on this. To date, there has been little attempt to understand how family and friends of those with mental health conditions understand, experience, and respond to the smoking behaviors of those they support. Although informal carers can provide a strong source of emotional and practical support for their relative, family members, and friends can lack awareness of available resources and fear social stigma.^82^ Therefore, they tend to adapt negatively to the individual’s smoking-related behavior,^82^ possibly providing an explanation for the dearth of literature within this population. However, such an absence of evidence highlights the need for further investigation into the role of informal support networks and the needs of informal carers, to increase their involvement in supporting attempts at changing smoking behavior.

The domain of intentions was also identified as influencing changes in smoking behaviors. In particular, the patient’s intentions and lack of interest in smoking cessation was identified as a barrier to engagement.^44,50,61^ Despite this, compelling evidence exists that indicates most people with mental health conditions do want to quit and intend to do so, and that smoking cessation interventions targeting this population are effective.^15^ It is important therefore, that fluctuations in motivation or intentions are not equated with wanting to disengage with support, but rather to allow the flexibility for individual’s to reengage when they wish to do so.^63^

Finally, our findings indicate that many of the factors identified by patients as barriers to smoking cessation or temporary abstinence related to the TDF domain of “emotion.” It was frequently reported that smoking was used as a coping strategy for everyday stressors and in relation to one’s mental health diagnosis.^52,56–58,62^ Similarly, psychosocial stressors have been implicated as risk factors for tobacco use in a range of populations, including people living with other health conditions,^83^ those from disadvantaged communities,^84,85^ those in the military,^86^ and those in the general population.^87^ Accordingly, this indicates the need to develop tailored interventions that target the identification and implementation of alternative coping strategies for individuals with mental health conditions.

### Limitations and Strengths

This review included studies comprising various methodological designs, and which included the perspectives of a range of stakeholders in variety of mental health settings. Even with this diversity of mental health settings, there appeared to be consistency in the findings across these contexts. Therefore, this review offers a comprehensive overview of the barriers and enablers to addressing smoking in mental health settings. However, a number of limitations should be acknowledged. This review only included studies from high-income countries, which limits the generalizability of our conclusions, since low- and middle-income countries may present different contextual, political, and economic barriers that were not explored. The data analyzed were obtained from the interpretation of the of the study findings from the article authors. Therefore, the potential for reporting bias cannot be excluded. Importantly, authors may have selectively reported findings on barriers and enablers, potentially drawing conclusions from those that aligned neatly with the research question, or which were perceived as controversial or interesting.

## Conclusion

Environmental context and resources, knowledge, social influences, intentions, and emotion are key factors influencing smoking cessation in mental health settings. Specific barriers to the delivery of intentions by MHPs include competing demands on time and resources and limited knowledge in relation to tobacco use and its links with mental health. Enablers to enhance patients’ engagement with smoking cessation support include tailored, personalized support, and the teaching of alternative coping strategies, and the inclusion of social networks with pro-quitting social norms. Targeting or exploiting these factors are more likely to result in successful interventions. Future research should explore the enablers and barriers to smoking cessation in low- and middle-income countries to identify contextual differences that may have an impact on smoking-related behaviors. Lastly, further research is required to seek the perception of the informal carers and patients’ social networks in relation to the support offered to address smoking in mental health settings.

## Supplementary Material

A Contributorship Form detailing each author’s specific involvement with this content, as well as any supplementary data, are available online at https://academic.oup.com/ntr.

ntac004_suppl_Supplementary_Table_S1

ntac004_suppl_Supplementary_Table_S2

ntac004_suppl_Supplementary_Table_S3

ntac004_suppl_Supplementary_Table_S4

ntac004_suppl_Supplementary_Table_S5

ntac004_suppl_Supplementary_Data
