# Supplementary material for: A Systematic Review of Mental Health Professionals, Patients, and Carers’ Perceived Barriers and Enablers to Supporting Smoking Cessation in Mental Health Settings
Source: Nicotine Tob Res. 2022 Jan 8;24(7):945–54. doi: 10.1093/ntr/ntac004 (PMC9199941; doi:10.1093/ntr/ntac004)
Supplement: ntac004_suppl_Supplementary_Table_S1 [file ntac004_suppl_supplementary_table_s1.docx]

**Supplementary Table 1.** Search terms

| **Population** | Patient; OR hospitalised patient; OR outpatient; OR inpatient; OR mentally ill patient; OR acutely ill; OR acute; OR community patient; OR hospital staff; OR hospital personnel; OR hospital worker; OR doctor, OR physician; OR psychiatrist; OR nurse; OR mental health nurse; OR psychiatric nurse; OR consultant; OR carer$; OR visitor$; OR service user; OR allied health professional; OR key worker; OR community worker; OR social worker; OR occupational therap* OR psychologist; OR therapist |
| --- | --- |
| **Setting** | Mental health; OR psychiat*; OR community mental health service*; OR mental health service*; OR acute mental health service*; OR mental health ward*; OR acute setting; OR secondary mental health; OR hospital*; OR outpatient mental health; OR inpatient mental health; OR psychiatry* service; OR psychiatric hospital; OR psychiatry* ward; OR admission; OR discharge; OR leave; OR readmission; OR clinic; OR acute OR ward; OR stay; OR unit; OR recover*; OR community. |
| **Smoking cessation** | Smoking; OR smoking prevention; OR tobacco prevention; OR cigarette# prevention; OR smoker# restrict#; OR cigarette# restrict#; OR tobacco restrict#; OR tobacco quit; OR tobacco abstain; OR smoking cessation; OR tobacco cessation; OR electronic cig*; OR e-cig*; OR vap* |
| **Barriers facilitators, and associated factors** | Barrier*; OR facilitator*; OR enable*; OR challenge*; OR implement*; OR implementation OR issue*; OR factor*; OR influence*, OR predict* |
