# Supplementary material for: A Systematic Review of Mental Health Professionals, Patients, and Carers’ Perceived Barriers and Enablers to Supporting Smoking Cessation in Mental Health Settings
Source: Nicotine Tob Res. 2022 Jan 8;24(7):945–54. doi: 10.1093/ntr/ntac004 (PMC9199941; doi:10.1093/ntr/ntac004)
Supplement: ntac004_suppl_Supplementary_Table_S2 [file ntac004_suppl_supplementary_table_s2.docx]

| Supplementary Table 2. Characteristics of included studies | | | | | | |
| --- | --- | --- | --- | --- | --- | --- |
| Author/Year | **Country** | **Study design** | **Method** | **Sample** | **Setting** | **Sample size** |
| Aschbrenner et al. (2019) | USA | Qualitative | Semi-structured interview | Patients | Community | 41 |
| Ballbe et al. (2012) | Spain | Quantitative - Descriptive | Cross-sectional survey | Clinical mangers | Inpatient; Outpatient | 186 |
| Bennett et al. (2015) | USA | RCT | Questionnaire, bio-chemical measures | Patients | Outpatient | 178 |
| Brown et al. (2015) | USA | Quantitative - Descriptive | Survey | Patients and psychiatrists | Community | 228 patients  28 Psychiatrists |
| Brunette et al. (2018) | USA | RCT | Survey; semi-qualitative interview | Patients | Outpatient | 81 |
| Burns et al. (2018) | Ireland | Qualitative | In-depth interviews; Focus groups | Patients | Community | 231 patients |
| Chen et al. (2017) | USA | Quantitative - Descriptive | Survey | Patients and range of staff | Community | 231 patients  142 staff |
| Dickerson et al. (2011) | USA | Qualitative | In-depth interviews | Patients | Outpatient | 78 |
| Guo et al. (2015) | Taiwan | Quantitative - Descriptive | Cross-sectional survey | Mental health nurses | Inpatient; Outpatient | 193 |
| Hall et al. (2006) | USA | RCT | Questionnaire | Patients | Outpatient | 322 |
| Himelhoch et al. (2014) | USA | Quantitative - Descriptive | Survey | Range of staff | Community | 95 |
| Huddlestone et al. (2018) | UK | Mixed-methods | Case note review; pharmacy documentation review; semi-structured interviews | Patients | Inpatient | 324 patients |
| Keizer et al. (2014) | Switzerland | Quantitative - Descriptive | Survey | Range of staff | Inpatient | 155 |
| Knowles et al. (2016) | UK | Qualitative | Semi-structured interview | Patients and staff | Community | 13 patients  3 staff members |
| Metse et al. (2016) | Australia | Quantitative - Descriptive | Survey | Patients | Community | 169 |
| Metse et al. (2016) | Australia | Quantitative - Descriptive | Survey | Patients | Inpatient | 754 |
| Metse et al. (2018) | Australia | Quantitative - Descriptive | Survey | Patients | Inpatient | 740 |
| Morris et al. (2009) | USA | Qualitative | Semi-structured focus group | Patients and a range of staff | Inpatient; Community | 62 patients  22 staff members |
| Okoli et al. (2017) | USA | Quantitative - Descriptive | Survey | Patients | Inpatient | 119 |
| Okoli et al. (2017) | USA | Quantitative - Descriptive | Survey | Range of staff | Inpatient | 195 |
| Ortiz et al. (2013) | USA | Quantitative - Descriptive | Survey | Directors and managers | Inpatient | Not available |
| Parker et al. (2012) | UK | Mixed-methods | Survey and structured qualitative data | Patients | Inpatient; Community | 2038 |
| Peckham et al. (2016) | UK | RCT | Structured baseline data | Patients | Community | 97 |
| Prochaska et al. (2013) | USA | Qualitative | Semi-structured interview | Patients and a range of staff | Outpatient | 14 patients  8 staff |
| Prochaska et al. (2014) | USA | RCT | Survey | Patients | Inpatient | 224 |
| Ratier-Cruz et al. (2020) | UK | Quantitative - Descriptive | Survey | Range of clinical and non-clinical staff | Inpatient; Outpatient; Community | 631 |
| Rogers et al. (2016) | USA | RCT | Survey | Patients | Community | 577 |
| Rogers et al. (2017) | USA | Quantitative - Descriptive | Survey | Patients | Inpatient | 384 |
| Rogers et al. (2018) | USA | Qualitative | Semi-structured interview | Range of staff | Community | 14 |
| Smith et al. (2019) | UK | Qualitative | Semi-structured interview / Focus group | Range of staff | Community | 36 |
| Wilson et al. (2019) | USA | Mixed-methods | Survey / Semi-structured interviews | Patients | Community | 13 |
