# Supplementary material for: A Systematic Review of Mental Health Professionals, Patients, and Carers’ Perceived Barriers and Enablers to Supporting Smoking Cessation in Mental Health Settings
Source: Nicotine Tob Res. 2022 Jan 8;24(7):945–54. doi: 10.1093/ntr/ntac004 (PMC9199941; doi:10.1093/ntr/ntac004)
Supplement: ntac004_suppl_Supplementary_Table_S3 [file ntac004_suppl_supplementary_table_s3.docx]

##### **Supplementary Table 2.** Assessment of risk of bias / quality assessment of included studies

| **MMAT – Qualitative studies** | | | | | |
| --- | --- | --- | --- | --- | --- |
| Author | *1.1. Is the qualitative approach appropriate to answer the research question?* | *1.2. Are the qualitative data collection methods adequate to address the research question?* | *1.3. Are the findings adequately derived from the data?* | *1.4. Is the interpretation of results sufficiently substantiated by data?* | *1.5. Is there coherence between data sources, collection, analysis and interpretation?* |
| Aschbrenner et al. (2019) | *Yes* | *Yes* | *Yes* | *Yes* | *Yes* |
| Burns et al. (2018) | Yes | Yes | Yes | Yes | Yes |
| Dickerson et al. (2011) | Yes | Yes | Yes | Yes | Yes |
| Knowles et al. (2016) | Yes | Yes | Yes | Yes | Yes |
| Morris et al. (2009) | Yes | Yes | Yes | Yes | Yes |
| Prochaska et al. (2013) | Yes | Yes | Yes | Yes | Yes |
| Rogers et al. (2018) | Yes | Yes | Yes | Yes | Yes |
| Smith et al. (2019) | Yes | Yes | Yes | Yes | Yes |

| **MMAT – RCT studies** | | | | | |
| --- | --- | --- | --- | --- | --- |
| Author | *2.1. Is randomization appropriately performed?* | *2.2. Are the groups comparable at baseline?* | *2.3. Are there complete outcome data?* | *2.4. Are outcome assessors blinded to the intervention provided?* | *2.5 Did the participants adhere to the assigned intervention?* |
| Bennett et al. (2015) | Yes | Yes | Yes | Unclear | Yes |
| Brunette et al. (2018) | Yes | Yes | Yes | Unclear | Yes |
| Hall et al. (2006) | Yes | Yes | Yes | Yes | Yes |
| Peckham et al. (2016) | Yes | Yes | Yes | Yes | Yes |
| Prochaska et al. (2014) | Yes | Yes | Yes | No | Yes |
| Rogers et al. (2016) | Unclear | Yes | Yes | No | Yes |

| **MMAT – Descriptive quantitative studies** | | | | | |
| --- | --- | --- | --- | --- | --- |
| Author | *4.1. Is the sampling strategy relevant to address the research question?* | *4.2. Is the sample representative of the target population?* | *4.3. Are the measurements appropriate?* | *4.4. Is the risk of nonresponse bias low?* | *4.5. Is the statistical analysis appropriate to answer the research question?* |
| Ballbe et al. (2012) | Yes | Yes | Yes | Yes | Yes |
| Brown et al. (2015) | Yes | Yes | Yes | Yes | Yes |
| Chen et al. (2017) | Yes | Yes | Yes | Yes | Yes |
| Guo et al. (2015) | Yes | Yes | Yes | Yes | Yes |
| Himelhoch et al. (2014) | Yes | Yes | Yes | Unclear | Yes |
| Keizer et al. (2014) | Yes | Yes | Yes | Unclear | Yes |
| Metse et al. (2016a) | Yes | Yes | Yes | Yes | Yes |
| Metse et al. (2016b) | Yes | Yes | Yes | Yes | Yes |
| Metse et al. (2018) | Yes | Yes | Yes | Yes | Yes |
| Okoli et al. (2017a) | Yes | Yes | Yes | Yes | Yes |
| Okoli et al. (2017b) | Yes | Yes | Yes | Yes | Yes |
| Ortiz et al. (2013) | Yes | Yes | Yes | Unclear | Yes |
| Ratier-Cruz et al. (2020) | Yes | Yes | Yes | Yes | Yes |
| Rogers et al. (2017) | Yes | Yes | Yes | Yes | Yes |

| **MMAT – Mixed-methods studies** | | | | | |
| --- | --- | --- | --- | --- | --- |
| Author | 5.1. Is there an adequate rationale for using a mixed method design to address the research question? | 5.2. Are the different components of the study effectively integrated to answer the research question? | 5.3. Are the outputs of the integration of qualitative and quantitative components adequately interpreted? | 5.4. Are divergences and inconsistencies between quantitative and qualitative results adequately addressed? | 5.5. Do the different components of the study adhere to the quality criteria of each tradition of the methods involved? |
| Huddlestone et al. (2018) | Yes | Yes | Yes | Yes | Yes |
| Parker et al. (2012) | Yes | Yes | Yes | Yes | Yes |
| Wilson et al. (2019) | Yes | Yes | Yes | Yes | Yes |
