# Supplementary material for: A Systematic Review of Mental Health Professionals, Patients, and Carers’ Perceived Barriers and Enablers to Supporting Smoking Cessation in Mental Health Settings
Source: Nicotine Tob Res. 2022 Jan 8;24(7):945–54. doi: 10.1093/ntr/ntac004 (PMC9199941; doi:10.1093/ntr/ntac004)
Supplement: ntac004_suppl_Supplementary_Table_S4 [file ntac004_suppl_supplementary_table_s4.docx]

**Supplementary Table 4.** Themes coded to the TDF domains, organised by perspective and influence (barrier/enabler/mixed)

| Theme | Context (Patient/MHP/Organisation/Mixed) | Influence  (Barrier/Enabler/Mixed) | Quotation | Summary of theme |
| --- | --- | --- | --- | --- |
| Environmental context and resources (n = 16 studies) | | | | |
| Task rich and time poor | Mixed | Barrier | Other barriers that were notably endorsed by psychiatrists were “lack of time (49%)” (Chen et al., 2016). | Limited time to support patients and requirement to prioritise conflicting demands. |
| Presence or absence of available support | Mixed | Mixed | From 2008 to 2011, fewer hospitals provided average smoking cessation care (56% and 35%, respectively, *p*=.05), and more hospitals provided good smoking cessation care (22% and 43%, respectively, *p*=.05), including follow-up and breadth of resources (Ortiz et al., 2013). | The availability and access to personalised and tailored support, with a range of different support strategies and an active, open and engaged recruitment approach. |
| Integration of services | Mixed | Mixed | There was a lack of resources to achieve the goals outlined in the smoke‐free policy, such as tobacco dependence treatment guidelines, bespoke recording instruments and NRT stock available to staff to implement smoking cessation support (Parker et al., 2012). | The presence or absence of policy and referral pathways and a coordinated approach to support, and the role of wider organisational networks. |
| Knowledge (n = 12 studies) | | | | |
| Lack of adequate knowledge on smoking cessation support | Mixed | Barrier | Some interactions around smoking that did occur were revealed to be ill-informed in relation to inaccurate advice (Burns et al., 2018). | Lack of knowledge regarding smoking cessation support, policies, and lack of knowledge regarding smoking in a mental health context; leading to the provision of misinformation. |
| Identified support needs | Patients | Mixed | Among service users, information and knowledge in relation to the effects of smoking; how to quit, and their own habits emerged as an important aspect of the programme. The knowledge was gained through leaflets and illustrations, teaching and filling in their own information: *“There was very good knowledge. I mean it was about the carbon monoxide, and about your breathing and about the habit and eh there was leaflets about if you give up smoking them, when you get the craving for a cigarette, how do you distract yourself from doing that, from having a cigarette, you know, do you listen to music, or have a meal or go for a walk, or bring the dog for a walk, or you know…I thought it was a good idea. Yeah it was very good knowing that. It was very good being taught that.”* (Burns et al., 2018). | The need for structured patient education to enhance knowledge and awareness of the benefits of quitting smoking, and knowledge on how to gain further support where required. |
| Social influences (n = 10 studies) | | | | |
| Influence of social network members | Patients | Mixed | Many participants reported that smoking was normative in many social contexts and socially acceptable among their peers and family members. The most commonly reported way network members got in the way of quitting smoking was by smoking cigarettes. As one participant said: *“It’s difficult, because everyone I know smokes”* (Aschbrenner et al., 2016). | Influence of social network members included social network members who smoked, smoking with network members, network members enabling smoking behaviours, and the impact of smoking norms, attitudes and behaviours of social network members. |
| Smoking among peers and smoking culture within a mental health context | Patients | Barrier | Patients reported frequently observing tobacco use among staff, with one person noting “they have to not smoke or they’re not a good example for me. If they smoke, they’ve got nothing to tell me” (Morris et al., 2009). | Smoking among peers and mental healthcare professionals, promoting a smoking culture within a mental healthcare context. |
| Intentions (n = 9 studies) | | | | |
| Lack of intention | Mixed | Barrier | Providers expressed belief that tobacco cessation for people with mental illnesses is unlikely to occur and not worth the effort, largely due to person's lack of motivation (Morris et al., 2009). | Patient and MHPs lack of intention to engage with and deliver smoking cessation support, and perception of patient’s lack of interest and intentions to engage. |
| Stability of intentions and stages of change | Patients | Enabler | Baseline measures of motivation (stages of change, thoughts about abstinence scales) and dependence (time to first cigarette) predicted abstinence status significantly (*P* < .05) (Prochaska et al., 2014). | Patient’s readiness to quit and the stability of their intentions to make a quit attempt. |
| Beliefs about capabilities (n = 8 studies) | | | | |
| Perceptions in relation to capability of delivering smoking cessation support | MHPs | Mixed | Higher perceived behavioural control in providing tobacco treatment scores were significantly associated with higher scores on providing brief interventions (Okoli et al., 2017). | The perceived behavioural control of staff and their perceived self-efficacy and confidence in being able to deliver appropriate smoking cessation support to smokers. |
| Perceptions in relation to patient’s capabilities | Mixed | Barrier | Lack of consistent determination/willpower was reported: *“It’s not having the willpower to stop doing something that’s very bad for you – if you have a mental health problem, it’s harder to give up smoking because I just haven’t got the mind, I haven’t got the willpower and I’m not able to make a decision to say I’m going to quit, and then just quit. I can’t, I find that difficult to do”* (Burns et al., 2018). | The perceived expectations regarding patients’ motivations and capabilities, and patients’ perceived capability to quit. |
| Emotion (n = 7 studies) | | | | |
| Coping mechanisms for stress | Mixed | Barrier | Coping mechanism for stress: Relapses were described in the context of acute stressors (e.g. health scares, family members falling ill, bereavements). In addition to acute stressors, smoking was also used to cope with the everyday stresses of life. Its role as a coping mechanism also emerged specifically in relation to mental health (e.g. anxiety, schizophrenia or psychosis) (Burns et al., 2018). | Relapses in the context of acute stressors, smoking used to cope with everyday stresses, and role as a coping mechanism in relation to mental health. |
| Lack of meaningful activities | Patients | Barrier | Two participants cited boredom and stress as challenges to managing their cravings and maintaining abstinence on the wards (Huddlestone et al., 2018). | Boredom, inactivity and filling a vacuum prompts individuals to smoke, and stress and boredom can challenge abstinence. |
| Reinforcement (n = 6 studies) | | | | |
| Incentives for quitting smoking | Patient | Enabler | Health and money as motivators: Physical health appeared to be the main motivation for quitting smoking among service users in general, and an important motivation for joining the programme. Overall, while health was the primary motivator, money also emerged, but was usually secondary (Burns et al., 2018). | Incentives for quitting smoking and/or making progress with smoking related behaviour goals, including health or financial benefits. |
| Smoking privileges as a behavioural reward | Patient | Barrier | Consumers often earned smoking privileges as a behavioural reward (Morris et al., 2009). | Barrier to smoking cessation or abstinence involves a smoking opportunity being offered as a behavioural reward during a smokefree stay. |
| Skills (n = 5 studies) | | | | |
| Levels of training and competency in delivering smoking cessation support | MHPs | Mixed | Other barriers notably endorsed by psychiatrists were “lack of training (62%)” (Chen et al., 2016).  Facilitators were resourceful in dealing with missed weeks and service users progressing through the programme at different paces. This included meeting more often and building individual work into group sessions to deal with some service users catching up on missed weeks. Some facilitators used the national quit line and quit website as an additional resource, used technology to support and empower attendees with low literacy, or had members who are former smokers come in to share advice (Burns et al., 2018). | Resourcefulness and competency of programme facilitators, and the levels of staff training in smoking cessation that would subsequently impact delivery. |
| Social/professional role and identity (n = 5 studies) | | | | |
| Perception of self | Mixed | Enabler | A trend approaching significance was found for seeing self as a smoker: participants who found it difficult to see themselves as a non-smoker were half as likely as those who could easily see themselves as non-smokers, to accept at least one cessation counselling call post discharge (odds ratio [*OR*]: 0.46, *p* = 0.05) (Metse et al., 2016). | Perception of self as a role model or as a non-smoker. |
| Perceived role of organisation/staff in addressing tobacco use | Mixed | Mixed | A fourth theme that was expressed during nine interviews is the culture or perception that mental health staff often view tobacco as a medical issue, not within a mental health provider’s scope of practice. Of note, the two providers on mental health leadership whom actively refused interview participation shared this perception when providing their reason for refusal (Rogers et al., 2018). | The extent to which one perceives addressing smoking is part of their job role/role within the organisation. |
| Beliefs about consequences (n = 4 studies) | | | | |
| Expectations and anticipation | Mixed | Enabler | The most important reasons for wanting to give up smoking were health related, with 97% of participants saying they wanted to give up because smoking was bad for their health and 95% saying that smoking made them less fit (Peckham et al., 2016). | Beliefs, outcome expectancies and characteristics of outcome expectancies of smoking abstinence and/or making a quit attempt, and understanding of and anticipation of past regrets and failures. |
| Goals (n = 4 studies) | | | | |
| Combination of smoking cessation goals with broader mental and physical goals | Patients | Enabler | Combining with other health initiatives: Combining smoking cessation with other broader mental and physical health initiatives seemed to be an effective approach. In some centres, service users seemed to naturally start making goals in other areas and link cutting down on smoking with healthier eating or exercise goals (Burns et al., 2018). | Combining goals or outcomes or end states that a patient is wanting to achieve. |
| Goal setting, maintenance and review | Patients | Enabler | Smokers in treatment for depression were more likely to be abstinent, to make at least one quit attempt if they had a more stringent abstinence goal (Hall et al., 2006). | The benefits of setting personalised goals; the benefits of reviewing these to ensure appropriateness and provide feedback on whether these are being obtained. |
| Memory, attention and decision processes (n = 2 studies) | | | | |
| Attentional, cognitive and motivational difficulties | Mixed | Barrier | Both the inpatient and community settings provided services to patients experiencing severe and enduring mental illness, and therefore significant barriers were related to attentional, cognitive and motivational factors, sometimes resulting in difficulties to engage and retain patients (Parker et al., 2012). | Illness-related factors that influence engagement, forgetting reasons for quitting that may influence engagement, and the challenges experienced by MHPs for delivering support due to attentional, cognitive and motivational difficulties. |
| Optimism (n = 1 study) | | | | |
| Need to maintain a positive attitude while making a cessation attempt | Mixed | Enabler | Positive expectation of mental health consumers’ ability to quit smoking was identified as a crucial prerequisite for successful smoking cessation efforts by both consumers and providers. Consumer and provider focus group participants noted that consumers need to maintain a positive attitude while making a cessation attempt as a negative outlook will assuredly lead to failure (Morris et al., 2009). | Having the positive outlook and confidence that desired goals will be attained. |
