# Supplementary material for: A Systematic Review of Mental Health Professionals, Patients, and Carers’ Perceived Barriers and Enablers to Supporting Smoking Cessation in Mental Health Settings
Source: Nicotine Tob Res. 2022 Jan 8;24(7):945–54. doi: 10.1093/ntr/ntac004 (PMC9199941; doi:10.1093/ntr/ntac004)
Supplement: ntac004_suppl_Supplementary_Table_S5 [file ntac004_suppl_supplementary_table_s5.docx]

**Supplementary Table 5.** Themes and sub-themes generated in the 13 identified domains of the TDF

| **Theme** | **Sub-theme** | **Sub-theme perspective (Patient/MHPs/Organisation)** | **Sub-theme influence**  **(Barrier/Enabler/Mixed)** |
| --- | --- | --- | --- |
| **Knowledge** | | | |
| Lack of adequate knowledge on smoking cessation support | Lack of knowledge regarding smoking cessation interventions and policies | MHPs | Barrier |
|  | Lack of appropriate knowledge to accurately inform patients | MHPs | Barrier |
|  | Limited knowledge and misconceptions surrounding support for quitting smoking | MHPs | Barrier |
|  | Lack of knowledge regarding smoking in a mental health context | MHPs | Barrier |
| Identified support needs | Inclusion of information on health effects | Patients | Enabler |
|  | Structured patient education | Patients | Enabler |
|  | Appreciation of greater mental health knowledge and awareness of MHPs | Patients | Enabler |
|  | Lack of knowledge of community resources for quitting smoking | Patients | Barrier |
| **Skills** | | | |
| Levels of training and competency in delivering smoking cessation support | Resourcefulness and competency of programme facilitators | MHPs | Enabler |
|  | Low levels of staff training in smoking cessation | MHPs | Barrier |
|  | Challenges of goal setting with patients | MHPs | Barrier |
| **Memory, attention and decision processes** | | | |
| Attentional, cognitive and motivational difficulties | Illness-related factors that influence engagement | Patient | Barrier |
|  | Forgetting reasons for quitting that influence engagement | Patient | Barrier |
|  | Challenges to delivering support due to attentional, cognitive and motivational difficulties | MHPs | Barrier |
| **Social/professional role and identity** | | | |
| Perception of self | Perception of self as role model | Patient/MHPs | Enabler |
|  | Perception of self as a non-smoker | Patient | Enabler |
| Perceived role of organisation/staff in addressing tobacco use | | Organisation/MHPs | Mixed |
| **Beliefs about capabilities** | | | |
| Perceptions in relation to capability of delivering smoking cessation support | Perceived behavioural control of staff | MHPs | Enabler |
|  | Limited self-efficacy and perceived ability to quit themselves | MHPs | Barrier |
| Perceptions in relation to patient’s capabilities | Negative expectations regarding patients motivations and capabilities | MHPs | Barrier |
|  | Limited self-efficacy and perceived ability to quit | Patients | Barrier |
| **Optimism** | | | |
| Need to maintain a positive attitude while making a cessation attempt | | Patients/MHPs | Enabler |
| **Beliefs about consequences** | | | |
| Expectations and anticipation | Expectations of abstinence and/or making a quit attempt | Patients | Enabler |
|  | Anticipation of consequences of smoking and quitting | Patients | Enabler |
|  | Understanding of and anticipation of past regrets and failures | Patients/MHPs | Enabler |
| **Intentions** | | | |
| Lack of intention | MHPs perception of lack of patient interest and intention | MHPs | Barrier |
|  | Patient lack of intention and interest | Patients | Barrier |
|  | MHPs lack of intention to deliver smoking cessation support | MHPs | Barrier |
|  | Absence of moderation of own smoking behaviour | MHPs | Barrier |
|  | Awareness and alignment with self, and/or organisation in promoting health | MHPs | Enabler |
| Stability of intentions and stages of change | Readiness to quit | Patients | Enabler |
|  | Stable intentions to make a quit attempt | Patients | Enabler |
|  | Stating intentions not to smoke | Patients | Enabler |
|  | Undertaking positive efforts that promote successful smoking cessation | Patients | Enabler |
| **Goals** | | | |
| Combination of smoking cessation goals with broader mental and physical goals | | Patients | Enabler |
| Goal setting, maintenance and review | Importance of goal setting with patient | MHPs/Patients | Enabler |
|  | Maintenance of goal setting | Patients | Enabler |
|  | Personal achievement of obtaining goals | Patients | Enabler |
|  | Flexibility of reviewing goals | MHPs/Patients | Enabler |
| **Reinforcement** | | | |
| Incentives for quitting smoking | Financial reward | Patients | Enabler |
|  | Positive impact on physical health | Patients | Enabler |
|  | Acknowledgement of progress made by a professional | Patients | Enabler |
|  | Positive feedback from a professional | Patients | Enabler |
|  | Impact of receiving CO feedback | Patients | Enabler |
|  | Impact of discussing CO feedback with MHP | Patients | Enabler |
| Smoking privileges as a behavioural reward | | Patients | Barrier |
| **Emotion** | | | |
| Coping mechanisms for stress | Relapses in the context of acute stressors | Patients | Barrier |
|  | Smoking used to cope with everyday stresses | Patients/MHPs | Barrier |
|  | Role as a coping mechanism in relation to mental health | Patients | Barrier |
| Lack of meaningful activities | Patient boredom | Patients/MHPs | Barrier |
|  | Patient occupation | Patients | Barrier |
|  | Patient inactivity | Patients/MHPs | Barrier |
|  | Patient filling inactive time prompts smoking | Patients | Barrier |
|  | Patient loss of interest and motivation due to lack of meaningful activities | Patients | Barrier |
|  | Boredom and stress challenges abstinence | Patients | Barrier |
| **Environmental context and resources** | | | |
| Task rich and time poor | Limited time to support patients | MHPs/Organisation | Barrier |
|  | Prioritisation of needs | MHPs/Organisation | Barrier |
|  | Limitations in clinic time | MHPs | Barrier |
|  | Competing demands on time and resources | MHPs | Barrier |
| Presence or absence of support offered | Presence of support | Patients/MHPs/Organisation | Mixed |
|  | Programme materials and format | MHPs/Patients | Enabler |
|  | Programme frequency and structure | MHPs | Barrier |
|  | Lack of referral resources | MHPs | Barrier |
|  | Availability of varied support | MHPs/Patients | Mixed |
|  | Availability of NRT | MHPs/Patients | Barrier |
|  | Locality of MH community settings | Patients | Enabler |
|  | Familiarity of settings and staff | Patients | Enabler |
|  | Limited offers of support | Patients | Barrier |
|  | NRT choices | Patients | Enabler |
|  | Personalised and tailored support | Patients | Enabler |
|  | Increased opportunities for seeking support | Patients/MHPs/Organisation | Enabler |
| Integration of services | Positive role of wider organisational networks | MHPs/Organisation | Enabler |
|  | Presence of policy and referral pathways | Patients/MHPs | Enabler |
|  | Absence of referral pathways | Patients/MHPs/Organisation | Barrier |
|  | Disjointed pathways | MHPs | Barrier |
|  | Presence of a coordinated approach to support | Patients/MHPs | Enabler |
|  | Adherence to policies | MHPs | Barrier |
| **Social influences** | | | |
| Influence of social network members | Smoking with network members | Patients | Barrier |
|  | Network members enable smoking behaviours | Patients | Barrier |
|  | Attitudes of social network members | Patients | Mixed |
|  | Smoking behaviours of social network members | Patients | Mixed |
|  | Quitting smoking with a network member | Patients | Enabler |
|  | Negative attitudes towards smoking by network members | Patients | Enabler |
| Smoking culture within a mental health context | Smoking with peers within the mental health context | Patients | Barrier |
|  | Smoking with MHPs within the mental health context | Patients | Barrier |
